# Clinical efficacy of continuous blood purification (CBP) in the treatment of severe acute pancreatitis with multiple organ dysfunction syndrome (MODS): A retrospective study

**DOI:** 10.1097/MD.0000000000045316

**Published:** 2025-10-24

**Authors:** Tianyi Mu, Songlin Yin, Rong Pu, Xuemei Yang, Chao Mai

**Affiliations:** a Emergency Department, Affiliated Hospital of North Sichuan Medical College, Nanchong, Sichuan, China; b Pediatrics, Affiliated Hospital of North Sichuan Medical College, Nanchong, Sichuan, China; c Pathology Department, Yingshan County People’s Hospital, Nanchong, Sichuan, China.

**Keywords:** continuous blood purification, inflammatory markers, multiple organ dysfunction syndrome, severe acute pancreatitis, survival rate

## Abstract

This study aims to evaluate the efficacy of continuous blood purification (CBP) in patients with severe acute pancreatitis (SAP) complicated by multiple organ dysfunction syndrome (MODS). The study analyzes the impact of CBP on organ function, inflammatory markers, length of hospital stay, and mortality. A total of 200 hospitalized patients who met the diagnostic criteria for SAP and MODS were included in this study. They were divided into the experimental group (CBP treatment group, n = 92) and the control group (standard treatment group, n = 108). The experimental group received CBP treatment, while the control group received standard medical supportive care. Propensity score matching was used to ensure baseline comparability between the 2 groups. Various statistical methods, including survival analysis and mediation effect analysis, were employed to assess the efficacy of CBP. At 24 hours posttreatment, the sequential organ failure assessment score was 7.5 ± 2.1 in the experimental group and 8.3 ± 2.4 in the control group (*P* = .02). At 72 hours and 7 days posttreatment, the sequential organ failure assessment scores in the experimental group were significantly lower than those in the control group (*P* < .001). Regarding inflammatory markers, C-reactive protein levels were 98.4 ± 37.5 mg/L in the experimental group compared to 110.5 ± 40.8 mg/L in the control group (*P* = .03). Interleukin-6 decreased to 42.6 ± 16.3 pg/mL in the experimental group, whereas it was 48.9 ± 19.2 pg/mL in the control group (*P* = .01). Tumor necrosis factor-α levels posttreatment were 52.4 ± 18.7 pg/mL in the experimental group and 67.1 ± 23.5 pg/mL in the control group (*P* = .002). The length of hospital stay was 22.7 ± 6.9 days in the experimental group and 20.8 ± 6.4 days in the control group (*P* = .02). The mortality rate was 15.3% in the experimental group and 26.4% in the control group (*P* = .04). The Kaplan–Meier survival curve analysis indicated that the survival rate in the experimental group was higher than in the control group (*P* = .021). CBP treatment significantly reduces systemic inflammatory responses, improves organ function, and lowers mortality in patients with SAP and MODS. Further studies are needed to optimize anticoagulation strategies in CBP to improve outcomes while minimizing the risk of complications.

## 1. Introduction

Severe acute pancreatitis (SAP) is a critical condition characterized by the sudden inflammation of the pancreas, which can rapidly progress to systemic inflammatory response syndrome (SIRS) and eventually lead to multiple organ dysfunction syndrome (MODS).^[1–3]^ The presence of MODS is one of the primary factors contributing to the high morbidity and mortality rates associated with SAP.^[4]^ Studies indicate that approximately 20% to 30% of SAP patients develop MODS, and these patients experience a significantly higher mortality rate. Thus, effective therapeutic strategies are essential to manage these patients and reduce adverse outcomes.^[5–7]^ Despite advances in supportive care, the management of SAP, particularly when complicated by MODS, remains a major clinical challenge.^[8,9]^ The pathophysiological mechanisms of SAP are complex, primarily involving the activation of pancreatic enzymes, local autodigestion of pancreatic tissue, and the subsequent systemic inflammatory response. SIRS exacerbates systemic inflammation, leading to dysfunction across multiple organs. Current treatment strategies mainly focus on supportive care, including fluid resuscitation, anti-infective therapies, and nutritional support. However, effective interventions for MODS remain limited, and traditional treatments have shown suboptimal outcomes, particularly in controlling inflammation and preventing organ failure.^[10]^

Continuous blood purification (CBP) has emerged as a potential therapeutic option for managing critically ill patients, including those with SAP complicated by MODS. CBP works by continuously removing inflammatory cytokines, metabolic waste products, and other harmful mediators from the bloodstream, thereby reducing systemic inflammation and stabilizing hemodynamics.^[10]^ Several studies have demonstrated that CBP can improve organ function, prevent further organ damage, and ultimately reduce mortality in SAP patients with MODS. Compared to intermittent blood purification, CBP provides continuous treatment over 24 hours, avoiding significant fluid and hemodynamic fluctuations, making it better suited for critically ill patients.^[7,9,11,12]^ However, the existing literature on CBP is limited by small sample sizes, lack of standardized protocols, and insufficient multicenter evidence, leading to uncertainties regarding its optimal use in clinical practice.

Given these considerations, we hypothesize that CBP can reduce systemic inflammation, enhance organ function, and improve the prognosis of SAP patients with MODS, thereby lowering mortality rates. The objective of this study is to evaluate the efficacy of CBP in patients with SAP complicated by MODS, particularly in comparison to standard clinical care. We aim to assess its effects on organ function, inflammatory markers, hospital length of stay, and mortality. This study seeks to provide more evidence-based data to guide the treatment of SAP with MODS, with the goal of optimizing therapeutic strategies and improving patient outcomes.

## 2. Materials and methods

### 2.1. Study population

This study was approved by the Ethics Committee of Affiliated Hospital of North Sichuan Medical College. This study included a total of 200 hospitalized patients with SAP complicated by MODS. These patients were treated at our hospital between January 2022 and January 2024 and met the diagnostic criteria for SAP and MODS. The main grouping criterion was whether or not the patients received CBP treatment, with the aim of comparing the efficacy of CBP in SAP patients with MODS. The treatment group received CBP to remove inflammatory mediators and modulate immune responses, thereby improving organ function. The control group received standard clinical care. Treatment group (CBP group, n = 92): patients treated with CBP. Control group (standard care group, n = 108): patients who did not receive CBP treatment and were managed with conventional therapy, including basic medical treatment such as fluid resuscitation and anti-infective therapy.

#### 2.1.1. Inclusion criteria

Confirmed SAP diagnosis: patients diagnosed with SAP according to the International Association of Pancreatology’s criteria, characterized by acute pancreatitis with organ dysfunction, local complications, or SIRS; complicated by MODS: defined as dysfunction in 2 or more organ systems, based on sequential organ failure assessment (SOFA) scores; age: patients aged between 18 and 80 years; early-stage patients: disease onset within 72 hours of admission (during the acute phase); informed consent: patients and their families provided informed consent for participation; complete clinical data: all required clinical data were available for analysis.

#### 2.1.2. Exclusion criteria

Preexisting severe underlying diseases: patients with severe comorbidities such as liver cirrhosis, chronic renal failure (nondialysis), or advanced malignancies that could impact treatment outcomes and prognosis; severe bleeding tendency: patients with significant coagulation disorders or other uncontrollable bleeding risks, making them ineligible for CBP treatment; uncontrolled septic shock: patients who remained in irreversible shock despite receiving intensive anti-infective therapy and vasoactive drugs; recent blood purification treatment: patients who had undergone blood purification or other hemofiltration procedures within 3 months prior to admission; pregnancy or breastfeeding: pregnant or breastfeeding women were excluded due to potential risks to the fetus or newborn associated with the treatment; coexisting central nervous system diseases: patients with severe central nervous system conditions such as cerebral hemorrhage or cerebral infarction were excluded.

### 2.2. Treatment methods

#### 2.2.1. Control group (standard treatment)

Patients in the control group received standard medical supportive care, without CBP treatment. The standard treatment was administered in accordance with established guidelines for the management of SAP and MODS. The following interventions were implemented:

(1) Fluid resuscitation: crystalloids, such as normal saline or lactated Ringer solution, were used for volume resuscitation to ensure adequate blood volume and organ perfusion. The goal was to maintain mean arterial pressure between 65 and 75 mm Hg and urine output >0.5 mL/kg/h.
(2) Nutritional support: enteral nutrition: early enteral nutrition was prioritized whenever the patient’s condition allowed, to reduce disruption of the gut barrier function and prevent bacterial translocation; parenteral nutrition: for patients unable to receive enteral nutrition, parenteral nutrition was provided to ensure adequate caloric and nutrient intake.
(3) Antibiotic therapy: broad-spectrum antibiotics were administered for early intervention in patients with confirmed or suspected infections. Antibiotic regimens were adjusted based on microbiological findings when necessary. Infection markers, such as C-reactive protein (CRP) and procalcitonin (PCT), were monitored to prevent antibiotic overuse.
(4) Pain and sedation management: Analgesics (e.g., morphine, fentanyl) and sedatives (e.g., dexmedetomidine) were used to control pain and anxiety, improving patient comfort. Dosages of analgesic and sedative medications were adjusted according to the patient’s clinical condition to avoid under- or overdosing.
(5) Glycemic control: insulin infusion was used to maintain blood glucose levels between 7.8 and 10.0 mmol/L, in order to prevent the adverse effects of hyperglycemia on patient outcomes.
(6) Hemodynamic support: for patients with unstable blood pressure, vasoactive drugs (e.g., norepinephrine) were administered to stabilize blood pressure. The dosage of vasoactive drugs was adjusted based on blood pressure changes and the patient’s hemodynamic parameters.
(7) Respiratory support: for patients with respiratory failure, mechanical ventilation was provided to maintain adequate oxygenation and respiratory function. Ventilator settings were adjusted according to the patient’s condition, aiming to maintain PaO2 > 60 mm Hg and SpO2 > 90%.
(8) Renal protection: Efforts were made to minimize the use of nephrotoxic drugs, maintain fluid and electrolyte balance, and prevent further deterioration of renal function.

#### 2.2.2. Experimental group: CBP treatment

In addition to receiving standard supportive care, patients in the experimental group underwent CBP treatment.

##### 2.2.2.1. CBP treatment protocol

Patients in the experimental group received CBP through continuous veno-venous hemofiltration or continuous veno-venous hemodiafiltration, which continuously removes inflammatory mediators, metabolic waste, and toxins, helping to reduce systemic inflammation and stabilize the internal environment.

(1) Filtration parameters: blood flow rate of 150 to 200 mL/min, and ultrafiltration rate of 20 to 25 mL/kg/h.
(2) Dialysate: used through a hemodiafilter with a flow rate of 1000 to 1500 mL/h.
(3) Anticoagulation: heparin or citrate was used; local or no anticoagulation for patients with bleeding risks.
(4) Treatment duration: continuous for 24 hours, typically 3 to 7 days, adjusted based on patient’s progress.

### 2.3. Observational indicators

(1) Demographic and baseline clinical characteristics: age, sex, disease duration, history of diabetes and hypertension.
(2) Basic treatment differences: use of mechanical ventilation; duration of mechanical ventilation; use of vasoactive drugs; duration of vasoactive drug use.
(3) Disease-related indicators: SOFA score: used to assess the severity of multiple organ dysfunction; APACHE II score: used to evaluate disease severity and predict prognosis; renal function (serum creatinine, μmol/L): changes in renal function were assessed by monitoring serum creatinine levels in both groups. Length of hospital stay and mortality rate.
(4) Inflammatory markers: CRP: monitored to evaluate changes in systemic inflammation, as a key inflammatory marker; interleukin-6 (IL-6): an inflammatory cytokine reflecting the level of inflammation in the body; tumor necrosis factor-α (TNF-α): an indicator of inflammation severity and treatment response; interleukin-1β (IL-1β): evaluated as an inflammatory marker; PCT: used to reflect the severity of bacterial infection and inflammation.
(5) Complications: sepsis; gastrointestinal bleeding; intra-abdominal hypertension; abdominal compartment syndrome (ACS); pancreatic encephalopathy. Ascites and pleural effusion: incidence of pleural effusion and lung infections were also evaluated.

### 2.4. Statistical methods

Various statistical methods were employed in this study to assess the efficacy of CBP treatment in patients with SAP complicated by MODS. First, propensity score matching (PSM) was used to ensure comparability between the 2 groups in terms of baseline characteristics. Continuous variables (e.g., length of hospital stay, inflammatory markers) were compared between groups using either the independent samples *t*-test or the Mann–Whitney *U* test, depending on data distribution. Categorical variables (e.g., complication incidence) were analyzed using the chi-square test. Survival curves were generated using Kaplan–Meier survival analysis, and the Cox proportional hazards regression model was used to evaluate the independent effects of CBP treatment and inflammatory markers on survival time. Within-group comparisons were performed using the paired samples *t*-test to assess changes before and after treatment. Mediation effect analysis was conducted to explore the role of inflammatory markers as mediators between CBP treatment and mortality. In addition, multivariable regression analysis was performed to control for potential confounding factors. Statistical significance was set at *P* < .05. All statistical analyses were conducted using SPSS (IBM CorporationCity, Armonk) and R software (Vienna, Austria).

## 3. Results

### 3.1. Baseline characteristics of patients

Before PSM, there were significant differences between the experimental group (n = 92) and the control group (n = 108) in several baseline characteristics, including age, SOFA score, APACHE II score, disease duration, proportion of patients with diabetes and hypertension, and length of hospital stay, as shown in Table 1. After matching, 72 patients were included in both the experimental and control groups, and no significant differences were observed in any baseline characteristics (*P* > .05). The average age of patients in the experimental group was 57.3 ± 11.2 years, while the control group had an average age of 56.8 ± 10.7 years. This matching ensured comparability between the 2 groups, eliminating baseline differences that could influence the outcomes, allowing for a more accurate evaluation of the efficacy of CBP treatment.

**Table 1:** Baseline information matching between the 2 groups.

Variables	Before matching				After matching			
	Experimental group	Control group	*t*/*X*^2^ value	*P* value	Experimental group	Control group	*t*/*X*^2^ value	*P* Value
n	92	108			72	72		
Age (yr)	58.5 ± 12.3	54.2 ± 10.8	2.62	.01	57.3 ± 11.2	56.8 ± 10.7	0.45	.65
Gender			4.42	.12			0.39	.53
Male	53	61			42	40		
Female	39	47			30	32		
SOFA score	9.5 ± 3.4	8.7 ± 2.9	2.15	.03	9.3 ± 2.6	9.1 ± 2.7	0.45	.65
APACHE II score	19.8 ± 5.1	17.3 ± 4.6	3.12	<.01	18.9 ± 4.8	18.4 ± 4.3	0.63	.53
Course of disease (d)	7.4 ± 3.8	6.9 ± 3.5	1.22	.03	7.2 ± 3.1	7.1 ± 2.9	0.34	.73
Diabetes			4.68	.04			0.05	.82
Yes	24	34			20	21		
No	68	74			52	51		
Hypertension			4.11	.05			0.09	.77
Yes	40	59			36	37		
No	52	49			36	35		
Renal function (creatinine μmol/L)	176.4 ± 50.2	165.3 ± 46.1	1.74	.08	172.1 ± 47.6	170.6 ± 45.3	0.29	.76
Mechanical ventilation use			0.15	.77			0.08	.78
Yes	58	64			42	40		
No	34	44			30	32		
Duration of mechanical ventilation (d)	4.6 ± 1.2	4.1 ± 1.5	2.58	.06	4.5 ± 1.3	4.4 ± 1.4	0.33	.74
Vasopressor use			0.67	.41			0.06	.81
Yes	60	71			38	37		
No	32	37			34	35		
Duration of vasopressor use (d)	5.2 ± 2.3	4.9 ± 2.1	0.91	.36	5.0 ± 2.1	4.9 ± 2.0	0.28	.78
Propensity score (*X̅*±*S*)	0.63 ± 0.14			.01	0.19 ± 0.07			.31

SOFA = sequential organ failure assessment.

### 3.2. Organ function recovery in both groups

A comparison between the 2 groups showed that the SOFA scores at admission were 9.3 ± 2.6 in the experimental group and 9.1 ± 2.7 in the control group, with no significant difference in the severity of illness at baseline (*U* = 0.26, *P* = .79), as shown in Table 2. This indicates that the initial severity of illness was statistically comparable between the 2 groups, ensuring the validity of the subsequent treatment efficacy comparison.

**Table 2:** SOFA time changes between the 2 groups.

Time point	Experimental group	Control group	*U* value	*P* value
Baseline	9.3 ± 2.6	9.1 ± 2.7	0.26	.79
24 h	7.5 ± 2.1	8.3 ± 2.4	2.32	.02
72 h	5.6 ± 1.9	7.1 ± 2.3	4.36	.001
7 d	3.2 ± 1.4	5.6 ± 2.0	6.95	<.001
*U* value	2.13	1.95		
*P* value	0.02	0.04		

SOFA = sequential organ failure assessment.

Furthermore, at 24 hours, 72 hours, and 7 days posttreatment, the SOFA scores in the experimental group were significantly lower than those in the control group. Within-group comparisons revealed a significant decrease in SOFA scores at each time point posttreatment in the experimental group (*U* = 2.13, *P* = .02). Although the control group also experienced a reduction in SOFA scores, the degree of improvement was smaller (*U* = 1.95, *P* = .04).

### 3.3. Comparison of hospital stay and mortality between the 2 groups

**Figure 1.**
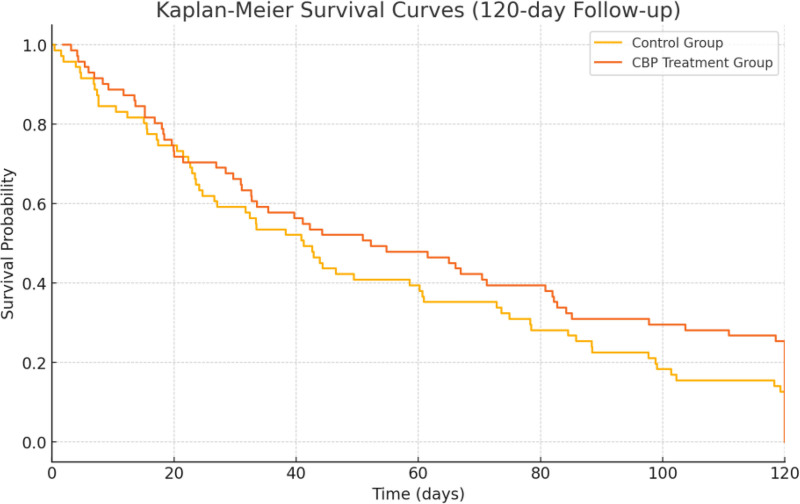
K-M survival curve between the 2 groups. CBP = continuous blood purification, K-M = Kaplan–Meier.

The comparison of hospital stay and mortality rates between the 2 groups is presented in Table 3. The average length of hospital stay in the experimental group (CBP treatment group) was 22.7 ± 6.9 days, while it was 20.8 ± 6.4 days in the control group (standard treatment group). The difference between the 2 groups was statistically significant (*U* = 2.34, *P* = .02). The mortality rate in the experimental group was 15.3%, compared to 26.4% in the control group, with the difference approaching statistical significance (χ^2^ = 3.76, *P* = .04). Additionally, the Kaplan–Meier survival curves for both groups, as shown in Figure 1, demonstrate a significant difference in survival rates (*P* = .021).

**Table 3:** Length of stay and mortality in the 2 groups.

Variable	Experimental group	Control group	*U*/χ^2^ value	*P* value
Length of hospital stay (d)	22.7 ± 6.9	20.8 ± 6.4	2.34	.02
Mortality rate (%)	15.3%	26.4%	3.76	.04

### 3.4. Inflammatory marker levels in both groups

We performed a comparative analysis of inflammatory markers between the 2 groups, as shown in Table 4. Before treatment, CRP levels in the CBP treatment group and the control group were 135.4 ± 48.2 mg/L and 132.6 ± 46.5 mg/L, respectively (*P* = .22), and IL-6 levels were 62.7 ± 19.8 pg/mL and 60.2 ± 18.6 pg/mL, respectively (*P* = .38), with no significant differences between the groups.

**Table 4:** Levels of inflammatory markers between the 2 groups.

Inflammatory marker	Pretreatment				Posttreatment			
	Experimental group	Control group	*U* value	*P* value	Experimental group	Control group	*U* value	*P* value
C-reactive protein (CRP, mg/L)	135.4 ± 48.2	132.6 ± 46.5	1.24	.22	98.4 ± 37.5	110.5 ± 40.8	2.15	.03
Interleukin-6 (IL-6, pg/mL)	62.7 ± 19.8	60.2 ± 18.6	0.87	.38	42.6 ± 16.3	48.9 ± 19.2	2.58	.01
Tumor necrosis factor-α (TNF-α, pg/mL)	85.3 ± 24.7	83.2 ± 25.3	1.15	.26	52.4 ± 18.7	67.1 ± 23.5	3.02	.002
Interleukin-1β (IL-1β, pg/mL)	47.5 ± 14.3	46.8 ± 13.7	1.1	.28	32.4 ± 11.5	40.3 ± 13.2	2.76	.006
Procalcitonin (PCT, ng/mL)	8.5 ± 2.1	8.2 ± 2.0	0.97	.33	3.9 ± 1.5	5.1 ± 2.0	2.41	.02

After treatment, CRP levels significantly decreased in the CBP treatment group to 98.4 ± 37.5 mg/L, compared to 110.5 ± 40.8 mg/L in the control group (*P* = .03). Similarly, IL-6 levels dropped to 42.6 ± 16.3 pg/mL in the CBP group, while the control group had IL-6 levels of 48.9 ± 19.2 pg/mL (*P* = .01), with both differences being statistically significant. Other inflammatory markers, such as TNF-α, IL-1β, and PCT, showed a similar trend, with the CBP treatment group demonstrating a significantly greater reduction compared to the control group.

### 3.5. Analysis of the impact of CBP on survival through inflammatory marker reduction

The mediation effect analysis results are presented in Table 5, showing that CBP treatment indirectly influences mortality through changes in inflammatory markers. Specifically, CBP treatment significantly reduced inflammatory marker levels (β_1_ = −0.45, *P* < .001), and changes in inflammatory markers were significantly associated with mortality (β_2_ = 0.38, *P* < .001), indicating that higher levels of inflammatory markers are associated with an increased risk of death.

**Table 5:** Mediation analysis result table.

Path	Estimate (*β*)	SE	*z*-value	*P*-value
CBP treatment → Inflammatory marker change	β_1_ = −0.45	0.12	−3.75	<.001
Inflammatory marker change → Mortality	β_2_ = 0.38	0.10	3.80	<.001
CBP treatment → Mortality	β_3_ = −0.20	0.14	−1.43	.15

CBP = continuous blood purification.

After controlling for changes in inflammatory markers, the direct effect of CBP treatment on mortality was no longer statistically significant (β_3_ = −0.20, *P* = .15). This suggests that the primary mechanism through which CBP treatment reduces mortality is by lowering inflammatory marker levels rather than directly affecting mortality itself. Therefore, it can be concluded that CBP treatment improves the prognosis of patients with SAP and MODS by reducing the inflammatory response.

### 3.6. Comparison of complications between the 2 groups

In this study, there were significant differences in the incidence of sepsis and gastrointestinal bleeding between the experimental and control groups (Table 6). The incidence of sepsis was 69.4% in the experimental group compared to 41.7% in the control group, with a statistically significant difference (*P* = .012), indicating that CBP treatment may offer an advantage in reducing the occurrence of sepsis. However, the incidence of gastrointestinal bleeding was 41.7% in the experimental group compared to 20.8% in the control group, with a significant difference (*P* = .045), suggesting that CBP treatment may be associated with a higher risk of gastrointestinal bleeding.

**Table 6:** Complication rates between the 2 groups.

Variable	Experimental group	Control group	*U*/χ^2^ value	*P* value
Sepsis	50 (69.4%)	30 (41.7%)	6.31	.012
IAH	20 (27.8%)	25 (34.7%)	0.43	.45
ACS	18 (25.0%)	12 (16.7%)	1.78	.18
Pancreatic encephalopathy	10 (13.9%)	5 (6.9%)	1.03	.31
Digestive tract bleeding	30 (41.7%)	15 (20.8%)	4.05	.045
Ascites	60 (83.3%)	55 (76.4%)	0.80	.37
Pleural effusion	50 (69.4%)	45 (62.5%)	0.68	.41
Lung infection	40 (55.6%)	35 (48.6%)	0.45	.50

ACS = abdominal compartment syndrome, IAH = intra-abdominal hypertension.

For other complications, such as intra-abdominal hypertension, ACS, pancreatic encephalopathy, ascites, pleural effusion, and lung infections, there were no statistically significant differences between the 2 groups (*P* > .05), indicating that the occurrence of these complications was comparable between CBP treatment and standard care.

## 4. Discussion

SAP is a life-threatening condition characterized by the sudden inflammation of the pancreas, which can progress to SIRS and subsequently lead to MODS.^[13]^ The presence of MODS is a major contributor to the high morbidity and mortality associated with this condition. Despite advancements in supportive care, the management of SAP, especially when complicated by MODS, remains challenging.^[10,14,15]^

CBP works by continuously removing inflammatory cytokines, metabolic waste products, and other harmful mediators from the bloodstream, thereby reducing systemic inflammation and stabilizing hemodynamic status. Several studies have suggested that CBP may help improve organ function, prevent further organ damage, and ultimately reduce mortality in critically ill patients.^[8,9]^ In this study, we evaluated the efficacy of CBP in patients with SAP complicated by MODS, comparing it to standard treatment.

The SOFA scores in the CBP group showed significant improvement compared to the control group at 24 hours, 72 hours, and 7 days after treatment. This suggests that CBP contributed effectively to stabilizing and enhancing organ function in SAP patients with MODS. The continuous removal of inflammatory mediators and the stabilization of hemodynamics by CBP likely played a key role in these improvements. Previous studies have also demonstrated the efficacy of CBP in reducing systemic inflammation and preventing further organ failure, consistent with our findings. CBP therapy significantly reduced levels of inflammatory markers such as CRP, IL-6, TNF-α, IL-1β, and PCT compared to the control group. The reduction in inflammatory cytokines indicates that CBP effectively mitigates the hyperinflammatory state observed in SAP, which is crucial for preventing the progression to MODS.^[16]^ The mediation analysis further supports this finding, showing that the beneficial effect of CBP on mortality was primarily mediated through a reduction in inflammatory markers. This highlights the potential mechanism of CBP in managing SAP (by controlling systemic inflammation, CBP indirectly improved survival outcomes). Although the CBP group had a longer hospital stay (22.7 ± 6.9 days) compared to the control group (20.8 ± 6.4 days), this difference should be interpreted with caution. The prolonged hospital stay in the CBP group is primarily attributed to the intensive nature of CBP therapy, which requires continuous monitoring and management over an extended period. Given that baseline severity was balanced between the 2 groups through PSM, the longer stay is more likely related to the treatment protocol rather than the complexity of the underlying disease. Thus, while the extended hospital stay is notable, it reflects the need for close monitoring during CBP therapy rather than a disadvantage of the treatment itself. The Kaplan–Meier survival analysis also demonstrated a significant survival advantage for patients receiving CBP treatment. These results underscore the importance of early and aggressive intervention in managing SAP complicated by MODS.

Despite the benefits observed, CBP therapy was associated with a higher incidence of gastrointestinal bleeding compared to the control group. This finding is consistent with previous studies suggesting that anticoagulation during CBP might increase the risk of bleeding. Conversely, CBP treatment was associated with a lower incidence of sepsis, which might be attributed to the reduced systemic inflammatory burden and improved immune regulation.^[6]^ The other complications, such as ACS, pleural effusion, and lung infection, showed no significant differences between the 2 groups, indicating that CBP did not notably increase the risk of these complications.

The findings of this study suggest that CBP therapy can be an effective adjunctive treatment for patients with SAP and MODS. By reducing systemic inflammation and improving organ function, CBP has the potential to improve outcomes in this critically ill population. However, careful consideration must be given to the risks of complications, particularly bleeding, and strategies to mitigate these risks should be explored. Further studies are warranted to optimize the anticoagulation protocols used during CBP and to identify specific subgroups of patients who may benefit the most from this therapy.

This study has several limitations. First, it was a retrospective analysis, which may introduce selection bias despite the use of PSM. Second, the sample size was relatively small, and larger multicenter studies are needed to confirm these findings. Third, the duration and timing of CBP were not standardized, which may have influenced the outcomes. Future research should focus on establishing optimal protocols for CBP duration and timing to maximize its benefits while minimizing risks.

## 5. Conclusion

CBP therapy appears to be a promising approach for managing SAP patients with MODS, primarily by reducing systemic inflammation and stabilizing organ function. While the therapy is associated with some risks, particularly bleeding, the overall survival benefit suggests that it can be a valuable tool in the treatment of critically ill SAP patients. To further validate its efficacy, a multicenter randomized controlled trial is needed to evaluate the impact of CBP on the 90-day survival rate. Additionally, future studies should focus on refining patient selection criteria, optimizing treatment protocols, and developing strategies to mitigate the risks associated with CBP therapy.

## Author contributions

**Conceptualization:** Tianyi Mu, Songlin Yin, Chao Mai.

**Data curation:** Rong Pu, Xuemei Yang, Chao Mai.

**Formal analysis:** Tianyi Mu, Songlin Yin, Chao Mai.

**Investigation:** Xuemei Yang, Chao Mai.

**Methodology:** Tianyi Mu, Songlin Yin, Chao Mai.

**Supervision:** Chao Mai.

**Validation:** Chao Mai.

**Visualization:** Chao Mai.

**Writing – original draft:** Tianyi Mu, Songlin Yin, Xuemei Yang, Chao Mai.

**Writing – review & editing:** Tianyi Mu, Songlin Yin, Xuemei Yang, Chao Mai.
